# Health literacy of trans and gender diverse individuals –a cross sectional survey in Germany

**DOI:** 10.1186/s12889-024-17823-4

**Published:** 2024-01-30

**Authors:** Rieka von der Warth, Mirjam Körner, Erik Farin-Glattacker

**Affiliations:** 1 Section of Health Care Research and Rehabilitation Research, Institute of Medical Biometry and Statistics, Medical Center – University of Freiburg, Faculty of Medicine, University of Freiburg, Hugstetter Str. 49, 79106 Freiburg, Germany; 2 Institute of Medical Psychology and Medical Sociology, University of Freiburg, Freiburg, Germany

**Keywords:** Health literacy, Transgender patients, Gender-diverse, Health promotion

## Abstract

**Introduction:**

To date, there has been little research on the general health literacy of trans and gender diverse individuals, even though previous research undermines the importance of good health literacy in this sample. The aim of the article is therefore to describe the general health literacy of trans and gender diverse individuals based on a German survey.

**Methods:**

In September 2022, a survey study was conducted in which health literacy was recorded using HLS-EU-16. Data will be presented descriptively; gender differences will be explored using a *Χ*^*2*^- test and a univariate analysis of variance (ANOVA).

**Results:**

Out of *N* = 223 participants, *n* = 129 individuals (57.8%) identified as non-binary; *n* = 49 (22.0%) identified themselves as male, while *n* = 45 (20.2%) identified as female. Mean age was 28.03 years. Overall, 26.4% of all the participants showed an inadequate health literacy, as proposed by the HLS-EU-16. In trend, health-related task related to media use were more often perceived as easy compared to the German general population.

**Conclusion:**

Individuals, who identify as trans and gender diverse may have a general health literacy below average compared to the German general population. However, tasks related to media use were perceived as easy, which might be a good starting point for health literacy related interventions.

**Trial registration:**

DRKS00026249*,* Date of registration: 15/03/2022.

## Background

The gender of trans and gender diverse individuals does not fully and/or constantly match their sex assigned at birth. They might identify with the opposite gender and therefore with a binary concept of gender; or they associate themselves with both, between or neither of the genders recognized by society (male/female), which is often referred to as gender-diverse [1]. Trans and gender diverse individuals face a great deal of stigma, discrimination and violence compared to cis-gender individuals [2, 3]. Stigma and discrimination are also prevalent within the health care system with doctors being known to have negative attitudes towards this community [4, 5] and transphobia playing an important role in the provision of health care services [6]. Furthermore, doctors are known to have little knowledge about the provision of trans and gender diverse specific care [5, 7]. As a result, trans and gender diverse individuals are known to avoid health care services due to the fear of mistreatment and harassment during consultations [8, 9].

Individuals, who encounter limited access to health care services, are known to seek health care information online [10]. In fact, trans and gender diverse individuals show a higher rate of search for health information online compared to cisgender individuals [11]. Furthermore, trans and gender diverse individuals are known to build online communities, where they inter alia share health information [12].

Both, finding information and appraising shared information are important factors within the concept of health literacy [13, 14]. Overall, health literacy is associated with the knowledge, motivation and competences to access, understand, appraise, and apply health information used to make decisions concerning the own health [14]. Low health literacy is associated with worse health outcomes in different samples [15]. Yet, little is known about the health literacy of trans and gender diverse individuals, even though trans and gender diverse individuals might need a higher degree of health literacy in order to navigate in a medical system that is based on a binary, biological gender system [16]. First qualitative studies therefore describe, that trans and gender individuals might have a good health literacy, as the participants themselves reported a good seeking behavior as well appraisal of information [17, 18]. On the contrary, one study assessed general health literacy using three single items as well as a validated eHealth literacy scale, and found a decreased level of health literacy of trans and gender diverse individuals compared to cisgender individuals [11]. As health literacy is known to affect health care access [19], having more knowledge on the health literacy of trans and gender diverse individuals is needed, as it might be another factor why this sample avoids health care next to stigma and discrimination. In fact, health literacy might be considered part of the individuals level in the integrated framework to understand health disparities in trans and gender diverse individuals described by Tebbe and Budge [20]. There, emotional and cognitive processes within the individual are described as possible protective and risk factor for the health risk of trans and gender diverse individuals.

Yet, we found no study describing the general health literacy of trans and gender diverse individuals, using a validated instrument, this paper seeks to explore and descriptively describe the health literacy of trans and gender diverse individuals in Germany. For this purpose, we used a validated instrument, making it easy to identify differences between the sample of interest and the general population. Gender differences in health literacy were tested in an exploratory way. Knowledge about the health literacy of trans and gender diverse individuals is needed to understand health disparities in this sample and to tailor specific health literacy interventions in order to ease the access to the health care system.

## Methods

Participant recruitment was conducted by the authors, working at the Medical Center – University of Freiburg. The ethical approval was granted by the Ethics Council of the University of Freiburg (Approval Number: 21–1609) and was registered in the German Clinical Trial Register (DRKS00026249).

### Study design and recruitment

Our study included individuals, which defined themselves as trans and/or gender-diverse. An official diagnosis by a doctor was not necessary. Furthermore, participants had to be of legal age of at least 18 years. No further inclusion or exclusion criteria were defined.

An online survey was conducted in September 2022. Recruitment was done via the RedCap platform [21, 22] using a snowball system. For this purpose, a study invitation was published on various social media platforms (Twitter, Facebook, etc.) describing all information for interested persons.

Before the interested individuals were forwarded to the survey, they were informed about the study in writing and their explicit consent was obtained. After consent was given, the inclusion criteria were queried. Both the declaration of consent and the inclusion criteria were programmed in such a way that the survey ended automatically, if you did not agree to participate or did not meet the inclusion criteria. Participants who completed the survey to the end received a €20 book voucher. This study was part of a bigger survey assessing health care needs and health care communication in trans and gender diverse individuals, which will be published elsewhere [23, 24].

### Instruments and analysis

To asses health literacy, the European Health Literacy Survey was used, in its 16 item short form (HLS-EU-Q16; 25). The items aim to assess the perceived difficulty of different tasks associated with health care and health promotion. Participants rate the task according to their perceived difficulty with “very easy” to “very difficult” on a four level Likert-scale. As part of the analysis, items are then dichotomized into “easy/1” and “difficult/0”, before creating a sum scale [25]. According to Röthlin, Pelikan and Ganahl [25] the sum score is then categorized into “sufficient” (13 to 16 points), “problematic” (9 to 12 points), and inadequate (1 to 9 points). Cronbach’s Alpha was α = 0.78 in this study. The instrument was adapted to gender-neutral language with permission of the original authors.

Additionally, sociodemographic data were recorded. We assessed, inter alia, sex, gender, and preferred pronouns. Additionally, we measured the belongings to different minorities as proposed by Szücs, Köhler [26].

To test gender differences in health literacy a *Χ*^*2*^- test and a univariate analysis of variance (ANOVA) was applied.

## Results

### Sample

Of 348 individuals who initially accessed the survey, 231 individuals completed the survey. 4 participants stated they neither identify with a binary nor a non-binary gender: these participants will be excluded from the analyses regarding the health literacy. Further 4 participants had to be removed from the data set due to ambiguous values, resulting in a final sample off 223 individuals. Mean age was 28.03 years (Std.-Deviation = 8.2 years). 57.8% of all participants identified themselves as non-binary, while 20.2% and 22.0% identified as female and male, respectively. Table 1 gives a more detailed overview of the participants.

**Table 1:** Sociodemographic Data of participants (*n* = 223)

	**All**		**Female (*n* = 45)**		**Male (*n* = 49)**		**Non-binary (*n* = 129)**	
	**Mean**	**Std. Deviation /Min–Max**	**Mean**	**Std. Deviation /Min–Max**	**Mean**	**Std. Deviation /Min–Max**	**Mean**	**Std. Deviation /Min–Max**
Age (years)	28.03	8.2 / 18–61	31.29	10.43	25.71	7.11	27.76	7.36
	**N**	**%**	**N**	**%**	**N**	**%**	**N**	**%**
Gender								
Female	45	20.2	45	100				
Male	49	22.0			49	100		
Non-binary	129	57.8					129	100
Sex assigned at birth								
Female	151	67.7	0	0	49	100	102	79.1
Male	72	32.3	45	100	0	0	27	20.9
Preferred pronouns								
She/her	53	23.8	41	91.1	1	2.0	11	8.5
He/him	54	24.2	0	0	46	93.9	8	6.2
They/them	37	16.6	1	2.2	0	0	36	27.9
Dey/den	12	5.4	1	2.2	0	0	11	8.5
Xier/xie	3	1.3	0	0	0	0	3	2.3
None/Addressing per name	37	16.6	2	4.4	2	4.1	33	25.6
Other	26	11.7	0	0	0	0	26	20.2
Minorities (multiple answers possible)								
Person of Color	2	0.9	0	0	1	2.0	1	0.8
Religious minority	8	3.6	2	4.4	2	4.1	4	3.1
Sexual minority	159	71.3	26	57.8	31	63.3	102	79.1
Gender minority	188	84.3	35	77.8	34	69.4	119	92.2
Person with disabilities	77	34.5	11	31.1	14	28.6	49	38.0
Other	24	10.8	6	13.3	3	6.1	15	11.6
Occupational status								
Employed without formal training	19	8.4	4	8.9	0	0	15	11.7
Employed with formal training	62	27.4	17	37.8	11	22.4	34	26.6
Self-employed	16	7.1	1	2.2	3	6.1	12	9.4
Tenured state employed	5	2.2	1	2.2	3	6.1	1	0.8
In pension	1	0.4	0	0	1	2.0	0	0
In training or in university	90	39.8	14	31.1	26	53.1	47	36.7
Not employed	33	14.6	8	17.8	5	10.2	19	14.8

### Health literacy of trans and gender diverse individuals

About 26% of all participants showed an inadequate health literacy according to the interpretation suggested by Röthlin, Pelikan and Ganahl [25], with male binary participants having the highest rate. At the same time, male binary participants showed the highest sum score (M: 11.3; SD: 3.4), due to the fact, that about 41% of all male participants also had a sufficient health literacy. All statistical testing, using the χ2 test and univariate ANOVA, returned non-significant, showing that there are no statistically significant gender differences in health literacy in this sample. Table 2 shows the details of the health literacy scores.

**Table 2:** Results of the HLS-EU-Q16 in categories and the sum score

	**All**		**Female**		**Male**		**Non-binary**		***p-Value***
	*N*	*%*	*N*	*%*	*N*	*%*	*N*	*%*	
**Sufficient**	77	34.5	16	35.6	20	40.8	41	31.8	
**Problematic**	87	39.0	16	35.6	13	26.5	58	45.0	
**Inadequate**	59	26.5	13	28.9	16	32.7	30	23.3	n.s
	*Mean*	*SD*	*Mean*	*SD*	*Mean*	*SD*	*Mean*	*SD*	
**Sum Score**	10.81	3.25	10.62	3.69	11.27	3.44	10.71	3.01	n.s

*n.s.* not significant, *SD* Std. Deviation

“sufficient” (13 to 16 points), “problematic” (9 to 12 points), inadequate (1 to 9 points) [25]

Regarding the individual items, the least participants rated items regarding the dimension “disease prevention/understanding information” as difficult. In contrast, most participants stated, that they perceived both, finding information on how to manage mental health problem and judging when to get a second opinion, as difficult. With both items, the portion of participants receiving that as difficult was about 69%. See Table 3 for an overview over all items.

**Table 3:** Percentage of participants receiving the health-related tasks as difficult or very difficult

*Area of interest*	**On a scale from very easy to very difficult, how easy would you say it is** **to: … **	**Very difficult or difficult**				
		Data from our study				Reference data [27]
		All participants (*n* = 223)	Female (*n* = 45)	Male (*n* = 49)	Non-binary (*n* = 129)	
		**N (%)**	**N (%)**	**N (%)**	**N (%)**	**%**
*Healthcare /Access information*	find information about symptoms of illnesses that concern you?	125 (56.1)	20 (44.4)	23 (46.9)	82 (63.6)	21.4
	find out where to get professional help when you are ill?	133 (59.9)	28 (62.2)	21 (42.9)	84 (65.6)	15.7
*Healthcare/ Understand information*	understand what your doctor says to you?	34 (15.2)	4 (8.9)	6 (12.2)	24 (18.6)	14.9
	understand your doctor’s or pharmacist’s instruction on how to take a prescribed medicine?	21 (9.5)	4 (8.9)	5 (10.2)	12 (9.4)	4.0
*Healthcare/ Appraise information*	judge when you may need to get a second opinion from another doctor?	154 (69.4)	29 (64.4)	34 (69.4)	91 (71.1)	42.8
*Healthcare/ Apply information*	use information the doctor gives you to make decisions about your illness?	95 (43.2)	16 (35.6)	17 (35.4)	62 (48.8)	29.1
	follow instructions from your doctor or pharmacist?	27 (12.1)	6 (13.3)	4 (8.2)	17 (13.2)	7.2
*Disease prevention/ Access information*	find information on how to manage mental health problems like stress or depression?	153 (69.2)	33 (73.3)	30 (62.5)	90 (70.3)	36.9
*Disease prevention/ Understand information*	understand health warnings about behavior such as smoking, low physical activity and drinking too much?	11 (4.9)	5 (11.1)	1 (2.0)	5 (3.9)	4.5
	understand why you need health screenings?	13 (5.8)	4 (8.9)	2 (4.1)	7 (5.4)	6.8
*Disease prevention/ Appraise nformation*	judge if the information on health risks in the media is reliable?	79 (35.4)	19 (42.2)	18 (36.7)	42 (32.6)	50.7
*Disease prevention/ Apply information*	decide how you can protect yourself from illness based on information in the media?	61 (27.6)	16 (35.6)	11 (23.4)	34 (26.4)	41.4
*Health promotion/ Access information*	find out about activities that are good for your mental well-being?	75 (33.85)	18 (40.0)	16 (33.3)	41 (31.8)	21.4
*Health promotion/ Understand information*	understand advice on health from family members or friends?	41 (18.5)	9 (20.5)	12 (24.5)	20 (15.5)	12.9
	understand information in the media on how to get healthier?	58 (26.2)	17 (37.8)	14 (29.2)	27 (21.1)	25.6
*Health promotion/ Appraise information*	judge which everyday behavior is related to your health?	62 (27.85)	13 (28.9)	12 (24.5)	37 (28.7)	13.9

Reference data is taken from Jordan and Hoebel [27], describing the health literacy in a representative German sample

## Discussion

In this paper, we sought to describe the health literacy of trans and gender diverse individuals in Germany. Using the HLS-EU-Q16, we found that almost two-thirds of all participants showed an inadequate or problematic level of health literacy. Compared to the German general population, this is an increased rate, as of to date only about 44–56% of the general population was reported to have an inadequate or problematic level [27–29]. Yet, our sample was relatively young and thus, should accordingly compared to a younger age group in the German general population. One study suggests that 44% in the rather broad age group of 18 to 39 year old German individuals show a problematic or inadequate level of health literacy [27]. In contrast, a more recent study classified individuals between the age of 18 and 29 years as vulnerable group, with a problematic and inadequate health literacy portion of 60% [29], which would be comparable to our study. Thus, it remains unclear if the health literacy of our sample is comparable to the health literacy of the German general population. Yet, the conclusion trans and gender diverse individuals might have a good health literacy as discussed in previous research [17, 18] must be rejected. Overall, the results undermine the results of Pho, Bakken [11], stating the health literacy of trans and gender diverse individuals is limited. This should be considered problematic, as previous research suggests, that trans and gender diverse individuals even need a higher degree of health literacy to navigate the health care system [16]. Consequently, the low degree of health literacy might be another reason, why trans and gender diverse individuals might not seek health care next to avoidance due to fear. It further might affect the health of trans and gender diverse individuals negatively.

Comparable to previous research on health literacy [27], we found no gender effects. However, on a descriptive level male participants had the highest level of health literacy in this study. At the same time, only about 30% of all non-binary participants showed a sufficient level of health literacy, being less compared to binary-gendered trans and gender diverse individuals in this study. This is in line with the literature, showing that non-binary trans and gender diverse individuals face additional barriers and burden [30].

Regarding the individual items, participants in this study received most of the described tasks more often as difficult compared to the German general population [27]. For instance, items concerning the access of information in healthcare were perceived as difficult by between 56–60% in this study, whereas only 16–21% of the German general population perceived them as such [27]. Additionally, tasks regarding communication with doctors, such as shared decision-making and understanding information given, were perceived as difficult by our sample. Doctor-patient-communication is an important enabler for patient-centered care [31], with the need to integrate patient preference being an essential part of successful communication [32]. Yet, many doctors do not have the required skills to provide patient-centered care to trans and gender-diverse individuals [33]. This might be, as training for doctors often consist of individual lectures on general LGBTIQA + health being given, with the teaching staff having various levels of competence for teaching about trans and gender diverse individuals health [34]. Thus, more teaching of doctors would be needed to meet the diverse health care needs of trans and gender diverse individuals.

Yet, participants perceived two tasks less difficult than other population groups: the appraisal and application of information regarding disease prevention. However, both items relate to media use. This might be of no surprise, as trans and gender diverse individuals have higher rates of searching health information online and sharing them in their communities [11, 12], probably because they might have special needs for information regarding gender affirming care [35]. However, much online information on gender affirming care might exceed the competence of trans and gender diverse individuals due to their complexity [36]. Yet, these results could be used to strengthen health literacy in trans and gender diverse individuals by developing specific online material and telehealth interventions to ease access to health care.

### Strengths and limitations

To our knowledge, this is the first study giving a broader insight into the health literacy of trans and gender diverse individuals. We used a widely validated assessment, which is culturally adapted for many countries, making a comparison easy. However, as we included no cisgender individuals, a direct comparison is not possible. In addition, upcoming research should further look into the relationship between health literacy and possible associated factors. For instance, education is known to be associated with health literacy with individuals having a higher education also showing better health literacy, while having a migration status is associated with worse health literacy [29]. Thus, health literacy in trans and gender diverse individuals should be investigated using an intersectional approach, analyzing the complex relationships of overlapping social identities. Yet, this approach was outside the scope of our study, as we sought to describe the overall health literacy as to date little is known about it in trans and gender diverse individuals. Furthermore, a bigger sample would be needed to assess these complex intersectional relationships.

Furthermore, about 58% of our sample identified as non-binary, which is not comparable with previous research, showing that about one fifth of trans and gender-diverse individuals identify outside a binary gender concept [37]. The reason for this gender ratio is probably the recruitment strategy via social media, where the call for participation has spread better in the subsample of non-binary individuals through the sharing of certain stakeholders. The recruitment strategy via social media might also be responsible for the low mean age of our sample, as social media is still more often used by younger individuals [38]. Thus, a sample bias should be assumed, even though recruiting using social media is assumed sufficient for trans and gender diverse individuals [39].

Lastly, even though we compared our data with a representative sample of the general German population, we do not claim generalizability for our study.

## Conclusion

Overall, trans and gender diverse individuals seem to have a low level of self-perceived health literacy. However, they show good rated competence regarding media use, which might be a good starting point to develop tele medical interventions to reduce barriers to health care. Yet, there is a lack of tele medical interventions for trans and gender diverse individuals [40] and validity of exciting interventions is limited [41]. Overall, information on health literacy as well as its impact on different health outcomes in the target sample remains understudied and should be further investigated.

## Data Availability

The datasets generated and/or analyzed during the current study are not publicly available due to data protection regulations, but are available from the corresponding author on reasonable request.
